# Impact of whole grain highland hull-less barley on the denaturing gradient gel electrophoresis profiles of gut microbial communities in rats fed high-fat diets

**DOI:** 10.1128/spectrum.04089-23

**Published:** 2024-05-15

**Authors:** Xuejuan Xia, Jing Lu, Xuanyu Chen, Lu Zhou, Yadong Huang, Shunjie Ding, Guannan Li

**Affiliations:** 1 School of Health Science and Engineering, University of Shanghai for Science and Technology, Shanghai, China; 2 Inner Mongolia Yili Industrial Group Co., Ltd, Hohhot, China; 3 Army Logistics University of PLA, Chongqing, China; 4 State Key Laboratory of Silkworm Genome Biology, College of Sericulture, Textile and Biomass Science, Southwest University, Chongqing, China; Chengdu University, Chengdu, China

**Keywords:** whole grain, fecal microbiota, denaturing gradient gel electrophoresis, nested PCR, high-throughput sequencing, taxonomic composition

## Abstract

Polymerase chain reaction-denaturing gradient gel electrophoresis (PCR-DGGE) is a traditional non-culture technique that can provide a fingerprint of the microbial community. In the field of gut microbiota analysis, PCR-DGGE still holds potential for development. In the present study, we utilized an improved nested PCR-DGGE approach targeting the V3 region of 16S ribosomal DNA to investigate the impact of whole grain highland hull-less barley (WHLB), a cereal known for its significant hypocholesterolemic effect, on the gut microbiota profiles of high-fat diet rats. Seventy-two male Sprague–Dawley rats were divided into four groups and fed a normal control diet, a high-fat diet, or a high-fat diet supplemented with a low or high dose of WHLB for 4 or 8 weeks. The results revealed that the dominant bands varied among different dose groups and further changed with different treatment times. The compositions of bacterial communities in feces and cecal content were similar, but the dominant bacterial bands differed. After performing double DGGE, extracting the bands, sequencing the DNA, and aligning the sequences, a total of 19 bands were classified under the Firmicutes and Bacteroidetes phyla, while two bands were identified as unclassified uncultured bacteria. The relative abundance of *Lactobacillus gasseri*, Uncultured *Prevotella* sp., and *Clostridium* sp. increased following the administration of WHLB. Illumina-based sequencing was employed to assess the reliability of DGGE, demonstrating its reliability in analyzing the dominant taxonomic composition, although it may have limitations in accurately detecting the alpha diversity of bacterial species.

**IMPORTANCE:**

While next-generation sequencing has overshadowed polymerase chain reaction-denaturing gradient gel electrophoresis (PCR-DGGE), the latter still holds promise for advancing gut microbiota analysis due to its unique advantages. In this study, we used optimized nested PCR-DGGE to investigate the gut microbiota profile of high-fat diet rats after administering whole grain highland hull-less barley. High-throughput sequencing was employed to validate the DGGE results. Our results proved the reliability of PCR-DGGE for analyzing the dominant taxonomic composition while also providing visual evidence of a notable relationship between the composition of cecal and fecal microbial communities, highlighting substantial differences in both richness and abundance.

## INTRODUCTION

Cardiovascular diseases (CVDs) have become a global public health concern, presenting significant challenges worldwide (1). The role of diet in the occurrence, development, and progression of CVDs is substantial, and adopting a healthy diet can effectively reduce the risk of CVDs (2–4). Unlike refined grain products, which primarily consist of the endosperm, whole grain foods contain abundant dietary fiber, micronutrients, vitamins, lignans, and phytochemicals, making them an essential component of a balanced and healthy diet (3, 5). Previous studies have reported the cholesterol-lowering effects of whole grain barley, attributing these beneficial effects to its bioactive components, especially dietary fiber and β-glucans (6–8). Highland hull-less barley, known as Qingke, is the most extensively cultivated grain crop on the Qinghai-Tibet Plateau. Due to its unique high-altitude growing environment, it possesses higher levels of β-glucans and dietary fiber (9, 10). Our previous research demonstrated that the consumption of whole-grain highland hull-less barley (WHLB) in the diet can reduce blood lipid levels in rats fed a high-fat diet (9). Currently, an increasing number of studies have indicated that the gut microbiota plays a role in regulating host processes such as fat absorption, transportation, storage, and metabolism. In a review by Tosh and Bordenave (11), they explored the primary mechanisms through which the consumption of whole grain oats, barley, and β-glucans reduces the risk of coronary heart disease. Notably, they emphasized the prebiotic effects of these components in modulating the gut microbiota to promote overall health. Therefore, gaining an understanding of how WHLB affects the gut microbiota can provide comprehensive insights into the regulatory mechanisms of WHLB on CVDs.

In the past few decades, research methods for studying the gut microbiota have undergone significant transformation. Early studies primarily relied on traditional culture techniques, where samples were placed in culture media to cultivate bacteria capable of growing under laboratory conditions (12). However, this method could only cultivate a small fraction of bacterial species, as the majority of microbial communities are unable to grow under *ex vivo* conditions. Subsequently, traditional non-culture techniques such as polymerase chain reaction-denaturing gradient gel electrophoresis (PCR-DGGE) (13, 14) and quantitative real-time PCR analyses (qRT-PCR) (15) have been employed to study microbial communities. However, these traditional molecular techniques have limitations. For instance, PCR-DGGE can only reflect information about a few dominant microbial populations in the samples and suffers from resolution errors (16). The qRT-PCR can only detect specific microorganisms (17). Recently, next-generation sequencing technologies have gained widespread application and have nearly overshadowed traditional techniques in microbial research. Next-generation sequencing offers advantages such as high throughput, high resolution, and comprehensiveness, as well as enabling various computational and statistical analyses (18–21). However, traditional techniques have found new ways to characterize the gut microbiota. The combination of traditional cultivation techniques and high-throughput sequencing technologies, termed culturomics, involves utilizing diverse cultivation conditions, media, and techniques to maximize the successful cultivation of various microbial species (22), enabling researchers to gain a better understanding of the diversity, functions, and interactions of microbial communities. Additionally, during high-throughput sequencing technologies, qRT-PCR can be utilized to validate sequencing data, quantify specific microbial taxa or genes, analyze gene expression, and detect low-abundance targets (23). What lies ahead for the future of PCR-DGGE?

PCR-DGGE offers several advantages, including cost-effectiveness, timely analysis, and ease of interpretation, making it a valuable tool for characterizing microbial communities (16). Recently, PCR-DGGE has emerged as a cutting-edge tool in the field of food traceability, enabling the differentiation of food products based on their quality and safety from production to consumption. It is a simple, rapid, inexpensive, reproducible, stable, and reliable method capable of handling a large number of samples in a single step (24, 25). While the limitations of DGGE primarily pertain to its taxonomic resolution, potential biases, band identification, and interpretation challenges, these factors should be taken into account during the analysis and interpretation of results (24, 26). Therefore, further exploration and improvement are necessary in terms of technological advancements, integration with other techniques, and data integration to meet the evolving demands of gut microbiota research. In this study, we aim to investigate the fingerprint of the gut microbial community of high-fat diet rats after WHLB administration by using the DGGE technique. To enhance the accuracy of DGGE analysis, we utilized nested PCR for improved sensitivity and specificity, optimized the denaturant concentration gradient and electrophoresis time, and employed secondary DGGE for band purification and validation. Moreover, we used high-throughput sequencing to verify the results of DGGE. The article helps us understand the impact of WHLB on the gut microbiota, thereby providing a comprehensive understanding of its regulatory mechanisms in CVDs. Additionally, it contributes to the improvement and application of DGGE in the analysis of gut microbiota.

## MATERIALS AND METHODS

### Animals and diets

Seventy-two specific pathogen-free male Sprague-Dawley rats at 4 weeks old with certificate number SCXK 2012-0005 (Chongqing) were provided by Chongqing Tengxin Biotechnology Co., Ltd. Whole grain highland hull-less barley (Tibetan *Hordeum vulgare* L. Zangqing 320) with high levels of β-glucans (5.77 ± 0.28 g/100 g), total dietary fiber (19.01 ± 0.54 g/100 g), and low fat content (1.03 ± 0.02 g/100 g) was obtained from Chongqing Jun Pro Food Co., Ltd. (China).

After adapting to the environment for 1 week, the rats were randomly divided into four groups (*n* = 18 per group): a negative control (NC) group fed a standard diet according to the American Institute of Nutrition AIN-93G (27), a blank control (BC) group fed a high-fat diet consisting of 100 g/kg lard and 10 g/kg cholesterol (28), a high-dose (HD) group fed a high-fat diet containing 489.5 g/kg WHLB replacing all of the cornstarch; a low-dose (LD) group fed a high-fat diet containing 100 g/kg WHLB. The specific formulas for each group’s diet are detailed in Table S1. Rats were housed in sterile stainless-steel cages, with three rats per cage and free access to food and water. The light was cycled every 12 h (8:00–20:00), with good ventilation, a temperature of 25°C ± 1°C, and a relative humidity of 45%–65%. To explore the time effect, half of the rats (nine rats from each group) were sacrificed after 4 weeks, while the remaining 36 rats were fed for an additional 8 weeks.

### Sampling of feces and cecum content

#### Sampling of feces

Fresh rat fecal samples were collected weekly for pH and moisture content measurements. Before the end of the experimental period (at 4 and 8 weeks), 200 ± 20 mg of fresh rat feces was collected after cleaning the rat anus with medical alcohol. The samples were then transferred to 2 mL sterile centrifuge tubes and stored at −80°C for subsequent microbial analysis.

#### Sampling of cecum content

After the experiment (at 4 and 8 weeks), rats were fasted for 12–14 h and anesthetized with ether before being sacrificed by decapitation. The cecum was removed, weighed after rinsing with ice-cold saline, opened, and the contents were collected by scraping. Under sterile conditions, 200 ± 20 mg of cecum content was accurately weighed and transferred to a 2 mL sterile centrifuge tube, which was then quickly transferred to −80°C for microbiota analysis. Approximately 0.5 and 0.2 g of the cecum content were taken for the determination of water content and pH, respectively.

### Moisture content and pH determination of feces and cecal content

Fresh fecal or cecal content samples (0.2 g) were placed in a 5 mL centrifuge tube, and 10 times the volume of distilled water was added. The mixture was then vortexed, and the pH value was immediately measured using a pH meter. Approximately 0.5 g of fresh fecal or cecal content samples were placed in a pre-weighed constant weight bottle and dried at 105°C until a constant weight was achieved. The formula for calculating moisture content was according to Shen et al. (29).

### Nested PCR-DGGE analysis

#### Nested PCR

##### DNA extraction

The genomic DNA of bacteria in feces and the cecal content of rats was extracted using a Stool DNA Kit (DP328, Tiangen Biotech Co., Ltd., Beijing, China) according to the manufacturer’s instructions. The extracted DNA was evaluated for purity and quality by 0.8% agarose gel electrophoresis and NanoDrop 2000 spectrophotometer (Thermo Scientific, Wilmington, MA, USA).

##### 16S ribosomal DNA PCR

The universal forward primer 27f (5′-AGA GTT TGA TCC TGG CTC AG-3′) and reverse primer 1492r (5′-GGC TAC CTT GTT ACG ACT T-3′) were used to amplify the 16S ribosomal DNA (rDNA) fragments (16). The PCR reaction was performed in a final volume of 50 µL consisting of 2 µL of DNA template, 0.2 µL of each forward and reverse primers, 5 µL of 10× PCR buffer (Mg^2+^ free), 5 µL of Mg^2+^ (255 mM), 2 µL of dNTP Mixture (2.5 mM each), 0.4 µL of Taq DNA polymerase (5 U/µL), and 35.2 µL of sterilized ddH_2_O, under the following conditions: 5 min at 94°C for initial denaturation, followed by 30 cycles of 40 s at 94°C for denaturation, 40 s at 55.5°C for annealing, and 1 min at 72°C for extension, and a final extension at 72°C for 7 min; storage at 4°C. The amplification product was detected by 1.5% agarose gel electrophoresis, and the target fragment was approximately 1,500 bp. The amplification product was stored at −20 ℃ for later use as a template for subsequent PCR.

##### Secondary amplification

The V3 variable region of 16S rDNA was amplified using primers 338f (5′-ACT CCT ACG GGA GGC AGC AG-3′) and 518r (5′-ATT ACC GCG GCT GCT GG-3′) (30). A 40-bp GC clamp (CGC CCG CCG CGC GCG GCG GGC GGG GCG GGG GCA CGG GGG G) was added to the 5′ end of the upstream primer 338f. The DNA amplification system included 2 µL of 16S rDNA template, 1 µL each of forward and reverse primers, 5 µL of 10× PCR buffer (Mg^2+^ free), 3 µL of Mg^2+^ (25 mM), 3 µL of dNTP mixture (2.5 mM each), 0.4 µL of Taq DNA polymerase (5 U/µL), and sterilized ddH_2_O was added to a final volume of 50 µL. The PCR reaction consisted of a touchdown protocol: 94°C for 5 min, 94°C for 40 s, 65°C–55°C for 1 min (−0.5°C/cycle), 72°C for 30 s, 20 cycles; 94°C for 40 s, 55°C for 1 min, 72°C for 30 s, 29 cycles; and 72°C for 7 min extension, followed by a hold at 4°C. The target fragment was about 200 bp. After detection by 2% agarose gel electrophoresis, the PCR product was stored at −20 ℃ for subsequent DGGE analysis.

### DGGE condition optimization

#### Denaturant concentration gradient

A perpendicular polyacrylamide gel (8%, wt/vol) was used to determine the optimal gradient of denaturant concentration ranging from 0% to 100% (see Table S2 for details). A total of 150 µL of mixed nested PCR amplification products from different samples were mixed with 30 µL of 6× loading buffer (Takara Bio, Inc., Dalian, China) and added to the gel wells. The gel was then electrophoresed on a DCode Universal Mutation Detection System (Bio-Rad, Hercules, CA, USA) for 3 h at a constant temperature of 60°C and a constant pressure of 150 V in 7 L of 1× TAE buffer (0.04 M Tris base, 0.02 M sodium acetate, and 1 mM EDTA, pH 8.0). After electrophoresis, the gel was removed and immersed in 100 mL of 3× GeneGreen staining solution (30 µL of 10,000× GeneGreen staining solution diluted with 10 mL of 1 M NaCl and 90 mL of dH_2_O, prepared freshly) for 30 min. After staining, the gel was washed with dH_2_O and observed using a Syngene GeneGenius Imaging System (Synoptics Ltd., Cambridge, UK).

#### Electrophoresis time

A denaturing gradient of 30%–50% (see Table S2 for the formula) was used based on the denaturant concentration gradient optimization result. The 125 µL of mixed nested PCR amplification products of different samples was mixed with 25 µL of 6× loading buffer, and 20 µL of the mixture was added every 30 min. The time progression was 120, 150, 180, 210, 240, and 270 min. After electrophoresis, staining and imaging were performed to determine the optimal electrophoresis time.

### DGGE analysis

#### Sample DGGE

The nested PCR amplification products (20 µL) of different samples from the same group were mixed with 4 µL of 6× loading buffer, and then the mixture was added to the sample wells. The gel was electrophoresed under a constant voltage of 150 V and a temperature of 60°C for about 220 min using an 8% acrylamide denaturing gel with a denaturant concentration gradient ranging from 30% to 50%. The gel was then stained, and the images were captured using the Syngene GeneGenius Imaging System. A sterile surgical blade was used to carefully excise the clear bands. Subsequently, each band was transferred into a sterile 1.5 mL centrifuge tube containing 30 µL of sterilized ddH_2_O and stored at −20 ℃ overnight for future analysis. The intensity of bands in DGGE profiles was analyzed using Image Lab 6.0 software (Bio-Rad) and quantified based on adjusted volume. Principal component analysis (PCA) was then conducted using the bands and their intensities to identify the relationships among different samples.

#### Secondary DGGE and band sequencing

The recovered bands were amplified again using 338f-GC and 518r primers. The PCR mixture consisted of 5 µL of DNA template, 0.2 µL each of forward and reverse primers, 5 µL of 10× PCR buffer (Mg^2+^ free), 3 µL of Mg^2+^ (25 mM), 3 µL of dNTP mixture (2.5 mM each), and 0.4 µL of Taq DNA polymerase (5 U/µL), with sterilized ddH_2_O to top up to a final volume of 50 µL. The amplification program was the same as that for the V3 region of the 16S rDNA. The amplified products were subjected to a secondary DGGE analysis (using the same method as for the first DGGE analysis) after detection by 2% agarose gel electrophoresis. For impure bands, multiple rounds of DGGE analysis were performed until only one band was present. The recovered bands were amplified again using 338f and 518r primers under the same conditions and program as the 16S rDNA PCR, with an annealing temperature of 56°C. The amplified products were detected by 2% agarose gel electrophoresis and sequenced by Shanghai Sangon Biological Engineering Technology and Service Co., Ltd. The sequencing results were submitted to the NCBI DNA database (http://www.ncbi.nlm.nih.gov) for similarity analysis using the BLAST software.

### High-throughput sequencing of cecal microbiota

The Illumina-based sequencing was performed on the cecal microbiota at week 8 to validate the DGGE results. The bacterial genomic DNA in the cecal contents was quantified using the Qubit 2.0 DNA Quantification Assay Kit (Thermo Scientific, Wilmington, MA, USA). The V3–V4 region of the bacterial 16S rRNA was amplified using the universal primers 341F (5′-CCT ACG GGN GGC WGC AG-3′) and 805R (5′-GAC TAC HVG GGT ATC TAA TCC-3′), with the forward primer incorporating a 6–8 bp barcode (31). After the initial amplification, a second amplification was performed using Illumina bridge PCR-compatible primers. Following completion of the PCR, the products underwent agarose gel electrophoresis and gel extraction. The extracted DNA was quantified, and high-throughput sequencing was carried out on the MiSeq platform (performed by Shenggong Biotechnology Co., Ltd.). In this process, six samples from the same group were combined into one sample, resulting in three samples per group. The raw sequence data have been deposited in the NCBI Sequence Read Archive Database under accession No. PRJNA315187.

After sequencing, paired-end reads were merged into a single sequence using FLASH software (32). Quality control and error correction were performed using PRINSEQ and MOTHUR software (33). The merged high-quality sequences were clustered into operational taxonomic units (OTUs) at a 97% similarity level using UCLUST software. Microbial analysis of the Chao1 index, ACE index, Shannon index, Simpson index, and coverage was conducted using the MOTHUR software. Species classification of the sequences was conducted using the RDP classifier software based on Bergey’s taxonomy, which includes domain, phylum, class, order, family, and genus. The distances between samples were visualized using the R programming package “pheatmap” based on Weighted UniFrac, presenting the heatmap of sample distances (34).

### Statistical analysis

Experiments were conducted in triplicate, except for the high-throughput sequencing. The data were presented as mean ± standard deviation. Normal distribution was assessed using the D’Agostino-Pearson test or Shapiro-Wilk test. Inter-group differences were assessed using one-way ANOVA with GraphPad Prism 8.0.1 (GraphPad Software Inc., CA, USA). The significance level for the test was set at *P* < 0.05.

## RESULTS

### Moisture content and pH of feces and cecal content

**Fig 1:**
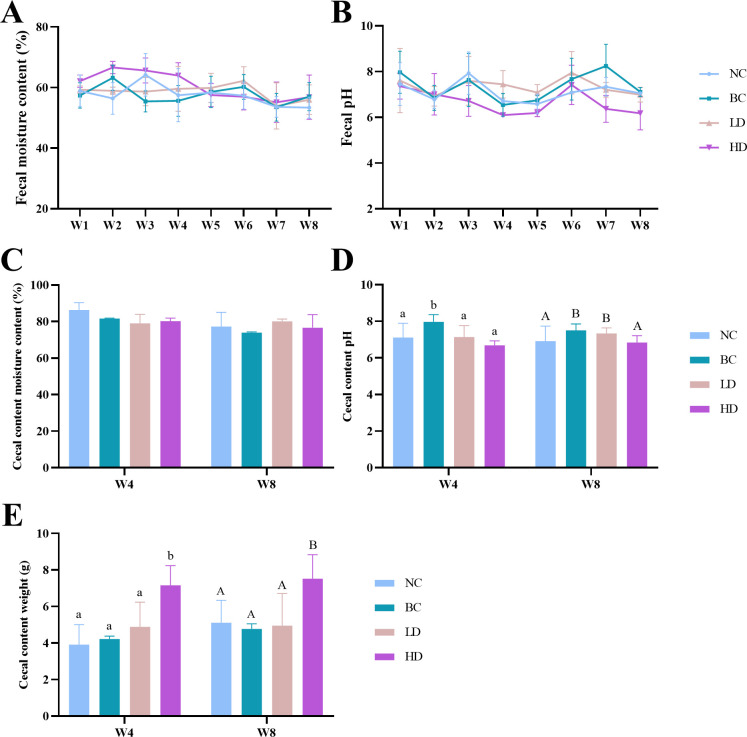
Effects of diets containing different doses of WHLB on the moisture content and pH of feces and cecal content in rats. The graphs are plotted as means with standard deviation, with 19 samples collected from week 1 to week 4 and 9 samples collected from week 5 to week 8 for fecal samples. For cecal content, nine samples were collected at both week 4 and week 8. One-way ANOVA was performed for statistical comparisons within groups. Values with different letters in the four groups are significantly different, with lower case letters indicating differences at week 4 and upper case letters indicating differences at week 8 (*P* < 0.05). The absence of a letter indicates no significant difference was observed. (A) Fecal moisture content. (B) Fecal pH. (C) Cecal content moisture content. (D) Cecal content pH. (E) Cecal content weight.NC, normal control group; BC, blank control group; LD, low-dose group; and HD, high-dose group.

The weekly influence of WHLB on rat fecal moisture content and pH is depicted in Fig. 1A and B. Both parameters showed a fluctuating pattern, and no significant differences were observed within the groups. The moisture content, pH, and weight of cecal content were measured at 4 and 8 weeks, and the results are presented in Fig. 1C through E. No significant differences in cecal content moisture were found among the groups (Fig. 1C). The cecum content pH (Fig. 1D) was significantly higher (*P* < 0.05) in the BC group compared to the other groups at 4 weeks. Additionally, at 8 weeks, the cecum content pH of both BC and LD groups was significantly higher (*P* < 0.05) than that of the NC and HD groups. Notably, the HD group showed significantly higher (*P* < 0.05) cecum content mass than other groups after both 4 and 8 weeks of feeding (Fig. 1E).

### Optimization of DGGE electrophoresis conditions

#### Nested PCR results

**Fig 2:**
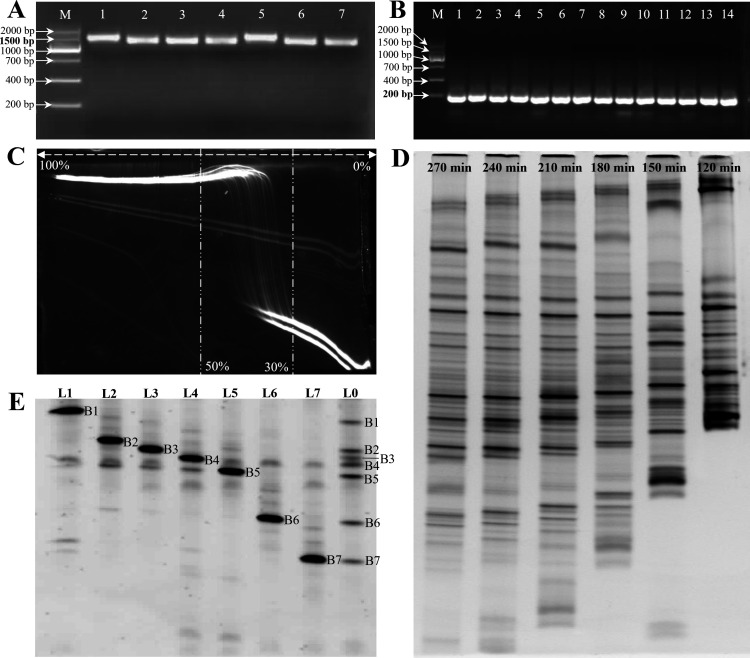
Nested PCR and DGGE condition optimization. (**A**) Agarose gel electrophoresis image of the 16S rDNA. (**B**) Agarose gel electrophoresis image of the V3 region of 16S rDNA gene. M, DNA Marker; lanes 1–14, representative samples. (**C**) Perpendicular DGGE profile of nested-PCR products. (**D**) DGGE profiles at different electrophoresis times. (**E**) Secondary DGGE profile of PCR products amplified from recovered DGGE bands. L1–L7, lanes for PCR products from recovered bands; L0, lane for the recovered band original sample; and B1–B7, representative recovered bands.

Nested PCR is known to reduce non-specific amplification and increase the concentration and purity of the target fragment (16). Figure 2A and B shows representative electrophoresis images of the amplification results of the 16S rDNA (1,500 bp) and the 16S rDNA V3 region (200 bp), respectively. Bright bands were observed in all lanes at the targeted positions, and the bands were clear without smearing or nonspecific amplification, indicating their suitability for subsequent analysis.

#### Denaturant concentration gradient

The denaturing gradient of vertical DGGE ran perpendicular to the electrophoresis direction and was employed to establish the appropriate denaturant gradient range for DNA fragments. Initially, the double-stranded DNA migrated along the electrophoresis direction. As the denaturant concentration increased, the double-stranded DNA gradually denatured, and the migration rate decreased. Different DNA fragments denatured at different concentrations, and within a certain range of denaturant concentrations, they exhibited multiple “S”-shaped curves. The range of the curve represented the appropriate denaturant gradient range (35). The denaturation of the nested PCR products was more effective in the denaturant gradient range of 30%–50%, as shown in Fig. 2C. Therefore, a denaturant concentration gradient of 30%–50% was chosen for the subsequent horizontal DGGE electrophoresis.

#### Electrophoresis time

Injecting samples at fixed time intervals into the gel could facilitate the exploration of the separation effect of bands at different electrophoresis times. As depicted in Fig. 2D, samples with varying electrophoresis times demonstrated different degrees of separation. When the electrophoresis time exceeded 240 min, some bands migrated off the gel. When the electrophoresis time was 210 min, the band separation was superior, but the time could be prolonged slightly. Therefore, an electrophoresis time of approximately 220 min was selected.

#### Secondary DGGE analysis

We performed a second-round DGGE analysis to confirm the location of the recollected bands and purify the recovered products further. As shown in Fig. 2E, a clear and bright band appeared in the amplification lane of each recollected band, corresponding to its position in the original sample.

### DGGE electrophoresis

#### DGGE profiles

**Fig 3:**
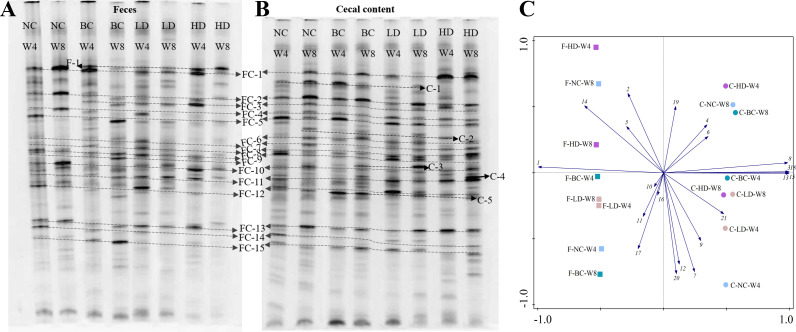
DGGE profiles and principal component analysis. (**A**) DGGE profiles and dominant bands observed in feces. (**B**) DGGE profiles and dominant bands observed in cecal content. Bands labeled with F, C, or FC indicate specific fecal, cecal content, or shared bands, respectively. (**C**) Principal component analysis of different samples based on the bands and their intensities. Samples labeled with a “F” or “C” indicate feces and cecal content samples, respectively.

The DGGE results of feces and cecal content bacterial profiles from rats in different groups after 4 and 8 weeks of feeding are depicted in Fig. 3A and B. The microbial compositions of feces and cecal content were comparable within the groups, characterized by a total of 15 shared bands (FC-1–FC-15), 1 fecal-specific band (F-1), and 5 cecal content-specific bands (C-1–C-5). These findings suggest that the fecal microbiota can partially reflect the composition of cecal microbial communities. However, an examination of the dominant bacterial bands in each sample revealed notable differences between feces and cecal content. For instance, in the HD group, there was a solitary prominent band (F-1) in feces at 8 weeks, whereas in the cecal content, several notable bands were observed, including FC-1, FC-8, FC-10, C-4, and FC-11. We quantified the band intensity (Table S3) and performed a PCA to visualize the differences between groups (Fig. 3B). Samples from different groups did not plot as distinct groups, but it was notable that samples from feces and cecal content formed two distinct clusters. Collectively, our data provide compelling evidence of a notable relationship between the composition of cecal microbial communities and the fecal microbiota. However, substantial differences exist in both richness and abundance between the two.

#### Band sequencing results

**Fig 4:**
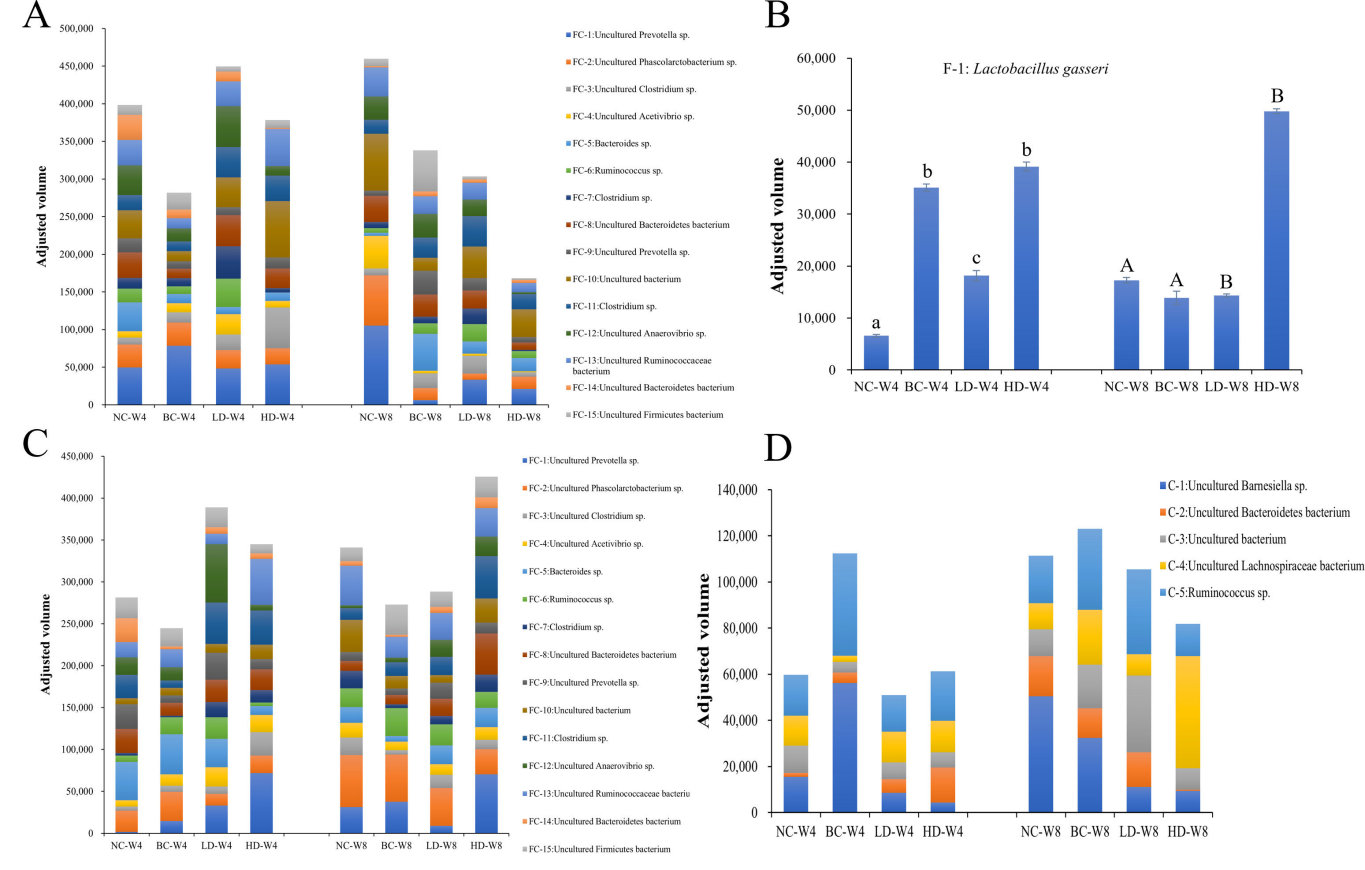
Band quantitation and sequence identification of bacteria in feces and cecal content. (**A**) Intensity and identification of dominant fecal bacterial bands. (**B**) Fecal-specific band intensity and identification in different groups. One-way ANOVA was performed for statistical comparisons within groups. Values with different letters in the four groups are significantly different, with lower case letters indicating differences at week 4 and upper case letters indicating differences at week 8 (*P* < 0.05). (**C**) Intensity and identification of cecal content dominant bacterial bands. (**D**) Intensity and identification of specific bands from cecal content.

After gel excision, secondary amplification, secondary DGGE, and non-GC amplification, selected bands were sequenced and identified with Blast alignment (Table S4). We generated composition histograms by utilizing their band intensity, as illustrated in Fig. 4. The fecal-specific bacterial community was represented by *Lactobacillus gasseri* (F-1), which exhibited higher abundance in the BC group at 4 weeks and the HD group at 4 and 8 weeks of dietary intervention. The cecal content-specific bacterial community included Uncultured *Barnesiella* sp. (C-1), Uncultured Bacteroidetes bacterium (C-2), Uncultured bacterium (C-3), Uncultured Lachnospiraceae bacterium (C-4), and *Ruminococcus* sp. (C-5). Referring to the taxonomic hierarchy of each bacterial community (36), the summary is presented in Table 1. Among the 21 sequenced bands, except for FC-10 and C-3, which could not be classified at the level of uncultured bacteria, the remaining 19 bands belonged to the phyla Firmicutes and Bacteroidetes.

**TABLE 1:** Classifications of bacteria identified by DGGE analysis^*a*^

Phylum	Class	Order	Family	Genus	Bands
Firmicutes	Bacilli	Lactobacillales	Lactobacillaceae	*Lactobacillus*	F-1
	Clostridia	Clostridiales	Acidaminococcaceae	*Phascolarctobacterium* sp.	FC-2
				*Anaerovibrio* sp.	FC-12
			Clostridiaceae	*Clostridium* sp.	FC-3, FC-7, FC-11
				*Acetivibrio* sp.	FC-4
			Lachnospiraceae	*Ruminococcus* sp.	FC-6, C-5
				−	C-4
			Ruminococcaceae	−	FC-13
	−	−	−	−	FC-15
Bacteroidetes	Bacteroidetes	Bacteroidales	Prevotellaceae	*Prevotella* sp.	FC-1, FC-9
			Bacteroidaceae	*Bacteroides* sp.	FC-5
			Porphyromonadaceae	*Barnesiella* sp.	C-1
	−	−	−	−	FC-8, FC-14, C-2
−	−	−	−	−	FC-10, C-3

^*a*^ “-” denotes unknown classification names.

Analyzing the composition of the cecal-content microbiota revealed that there were certain differences among the groups of rats at both 4 and 8 weeks of feeding, indicating that the microbial communities changed over time with feeding. Moreover, there were significant differences in dominant bacterial communities between the LD group and the HD and BC groups, suggesting that different doses of WHLB in high-fat diet-fed rats can have varying effects on the cecal microbiota. In comparison to the NC group, the BC group of rats showed the presence and dominance of C-5 (*Ruminococcus* sp.) at 4 and 8 weeks of dietary intervention. In contrast, the HD group of rats exhibited a noticeable dominance of FC-1 (Uncultured *Prevotella* sp.) at 4 and 8 weeks of feeding, with FC-8 (Uncultured Bacteroidetes bacterium) and C-4 (Uncultured Lachnospiraceae bacterium) showing significant dominance at 8 weeks of feeding. FC-11 (*Clostridium* sp.) was prominently dominant in both the LD and HD groups, particularly at 8 weeks of feeding. Analyzing the taxonomic hierarchy of bacterial communities in each group revealed that the dominant bacterial communities in all groups consisted of members from both the Bacteroidetes and Firmicutes phyla simultaneously.

### High-throughput sequencing verification

We conducted 16S metagenomics sequencing on the cecal content at week 8. The obtained reads ranged from 23,441 to 153,833 for different samples, with an average length of approximately 420 bp. The coverage in each sample exceeded 90%, ensuring a reliable estimation of the intestinal microbiota.

**Fig 5:**
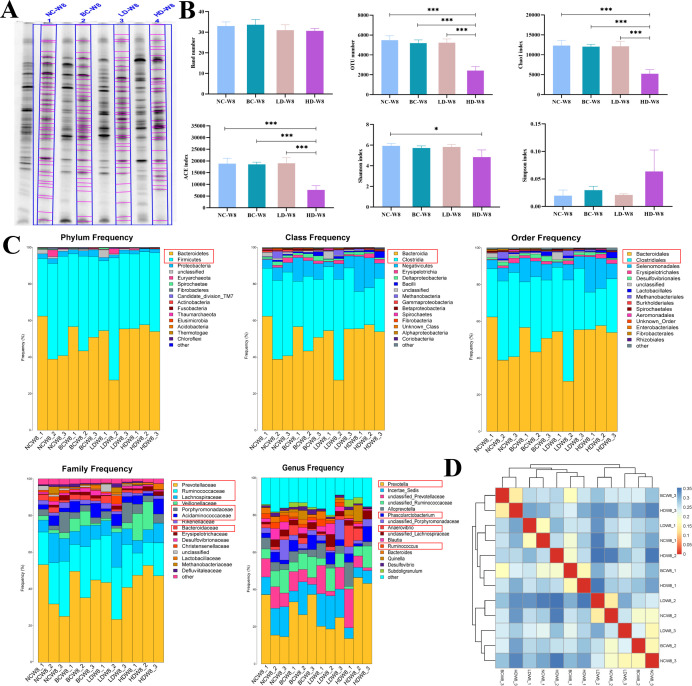
High-throughput sequencing verification of DGGE result using cecal content at week 8. (**A**) DGGE profiles at week 8 of cecal content with automatic band detection. (**B**) Analysis of band number by DGGE and alpha diversity indexes using Illumina-based sequencing. Statistical comparisons within groups were performed using one-way ANOVA. *P < 0.05 and ***P < 0.001. (**C**) Frequency of species classification in the sequences, including domain, phylum, class, order, family, and genus. The species that were also detected by the DGGE method were highlighted in red squares. (**D**) Heatmap of the Weighted UniFrac distance matrix illustrating the distances between samples.

We juxtaposed the detected band number of DGGE with the alpha diversity of 16S metagenomics sequencing for comparison in Fig. 5A and B. The band number of the DGGE profile showed no significant difference. However, the number of OTUs, Chao index, ACE index, and Shannon index exhibited significant variations within the samples, indicating a decreased alpha diversity in the HD group. These results indicate that the DGGE method may not be highly accurate in detecting the alpha diversity of bacterial species within the samples.

Encouragingly, in terms of the variations in species composition among different samples, it was observed that the majority of dominant bacterial communities detected by the DGGE method (Table 1) were also identified in the 16S metagenomics species classification results (Fig. 5C). Notably, the top two classifications determined by 16S metagenomics were consistently detected by DGGE at the phylum to family taxonomic levels. The sample distances depicted in Fig. 5D, representing the high-throughput sequencing results, showed no discernible clustering between groups, which aligned with the findings from the DGGE results (Fig. 3C). These findings together suggest that DGGE is reliable for analyzing the dominant taxonomic composition.

## DISCUSSION

The 2015–2020 Dietary Guidelines for Americans recommend that at least half of all consumed grains should be whole grains (37). Studies have demonstrated that increased whole grains consumption can reduce the risk of cardiovascular disease (38). Hence, in this study, the high-dose WHLB group was administered a high-fat diet containing 489.5 g/kg whole-grain barley replacing all of the cornstarch. Meanwhile, the low-dose group was fed a high-fat diet containing 100 g/kg WHLB, which was lower than the recommended level. The effects of WHLB on cecal weight, as well as the pH and moisture content of fecal and cecal samples, were determined. The results revealed a significantly higher cecal weight (*P* < 0.05) in the HD group compared to the other three groups. The cecum, being the widest, shortest, and most diverse part of the large intestine, contains a substantial amount of undigested material from the small intestine (39), corresponding to the high dietary fiber content of WHLB. The pH measurements of feces and cecal contents indicated an increase in pH values after consuming a high-fat diet. However, the high-dose WHLB diet significantly reduced the pH. A lower intestinal pH is beneficial as it inhibits the growth of putrefactive bacteria and holds important implications for the prevention and treatment of intestinal disorders (18). Similar to our study, Shen et al. (29) demonstrated that different doses of oat and barley β-glucans significantly reduced the fecal pH in rats. Additionally, Shen et al. (29) also found that β-glucans significantly increased the fecal moisture content in rats. In our study, no significant differences in moisture content were observed among the cecal contents of the different groups. Fecal moisture content in rats fluctuated over time, with the HD group exhibiting slightly higher moisture content than the other three groups in the first 4 weeks of feeding. However, no significant differences were observed among the groups after 5 weeks. This suggests that the WHLB diet had no significant effect on the moisture content of intestinal contents in rats fed a high-fat diet, possibly due to the water-holding capacity of the components in WHLB and the metabolism of the intestinal microbiota (40, 41).

The profiles of the intestinal microbiota in fecal and cecal content were analyzed using the DGGE technique. Studies often rely on fecal samples to investigate the changes in the gut microbiota of the organism (26). Our results indicated that the composition of the fecal and cecal microbiota was similar among the rat groups, but there were differences in prominent bacterial bands. This could be attributed to the fact that the majority of intestinal microbiota consists of obligate anaerobic bacteria, which are inhibited during fecal excretion, while a small number of facultative anaerobic bacteria increase in abundance with higher oxygen concentration (42, 43). Hence, it is advisable to minimize sample exposure to air during the collection of intestinal microbiota samples. Moreover, studies have revealed that microbiota patterns in cecal contents are different from those in feces because of the specific ecological conditions (44, 45). Therefore, although fecal samples are commonly used to analyze the gut microbiota, they do not fully reflect the abundance and composition of the gut microbiota. Analysis of the DGGE profiles of the microbiota in different rat groups revealed changes in microbial composition over time, and different doses of WHLB exhibited varying effects on the cecal microbiota of rats fed a high-fat diet. The influence of feeding time on the intestinal microbiota is gradually gaining attention (46). For instance, Murphy et al. (47) discovered that the intestinal microbiota of mice changed with feeding time after a high-fat diet, suggesting the need to consider this factor in future clinical research designs. It was observed that the HD group displayed more prominent bands after 8 weeks of the diet, indicating that a high-dose WHLB diet selectively enriched the gut microbiota in rats, with certain bacterial populations becoming more dominant. These dominant populations could restrict the growth of other bacterial populations (48), resulting in a decrease in overall species diversity, as verified by high-throughput sequencing. Consistently, Zhong et al. (49) investigated the effect of whole grain barley on cecal microbiota in high-fat diet rats and reported that the alpha diversity in the barley group was lower than that in the control group. Additionally, this corresponds to the reduced pH observed after the WHLB diet, as lower pH levels may inhibit the growth of certain bacteria (18).

Recovered band classification queries revealed that out of the 21 isolated bands, 19 were classified under the phyla Firmicutes and Bacteroidetes, while two bands remained unclassified. The two dominant phyla were further confirmed by the results of 16S metagenomics species classification. This finding is consistent with the reports of Schwiertz et al. (17) and Zhang et al. (50). The specific band unique to fecal samples (F-1) was identified as *Lactobacillus gasseri*, and it exhibited higher abundance in the HD group after 4 and 8 weeks. Although this bacterium was not identified as a dominant microbial population in the cecal contents, it is likely due to its ability to grow and reproduce in aerobic environments (51, 52). However, the variations in its abundance in the feces of different rat groups can still reflect its varying abundance in the intestinal tract. *Lactobacillus gasseri* is a probiotic of human origin known for its bile acid tolerance and its ability to improve the intestinal environment (51). These findings are consistent with our previous study, which demonstrated that WHLB increases the excretion of bile acids in the small intestinal content (9). In addition, the WHLB diet increased the relative abundance of *Prevotella* sp. (FC-1) and *Clostridium* sp. (FC-11). Similar to our study, Kovatcheva-Datchary et al. (53) reported an increase in the abundance of *Prevotella* in the intestine with barley intake. Research has shown that the increase in *Prevotella* was associated with dietary fiber intake (54, 55). Therefore, the significant increase in *Prevotella* may be due to the high dietary fiber content in the WHLB. Gérard (56) reported that bacteria of the *Clostridium* genus were involved in bile acid transformation in the gut. Therefore, the above results suggest that WHLB may regulate rat lipid metabolism by selectively enriching the abundance of specific bacteria, including *Lactobacillus gasseri, Prevotella*, and *Clostridium*, in the intestinal tract.

To enhance the accuracy of DGGE analysis, we employed nested PCR to enhance the sensitivity and specificity of the PCR, while minimizing non-specific amplification. Additionally, we optimized the denaturant concentration gradient and electrophoresis time to generate optimized DGGE profiles. Furthermore, we employed secondary DGGE to purify and validate the positions of the retrieved bands. These approaches collectively contribute to improving the accuracy of DGGE analysis results. The reliability of DGGE in analyzing the dominant taxonomic composition was confirmed through high-throughput sequencing verification. The DGGE method exhibited consistency with the 16S metagenomics species classification results, as it consistently detected the dominant bacterial communities. However, it is important to note that DGGE may not provide highly accurate results for assessing the alpha diversity of bacterial species within the samples. This limitation arises from inconsistencies between the number of bands observed in the DGGE profile and the alpha diversity indexes.

### Conclusion

Our results revealed that different feeding doses of WHLB have different effects on the cecal microbiota of rats fed a high-fat diet, and the composition of the microbiota changes with feeding time. A high dose of WHLB increases cecum bulk and reduces the pH of cecal content. Additionally, it may regulate rat lipid metabolism by selectively enriching the abundance of specific bacteria in the intestinal tract. Comparative analysis demonstrated that the microbial compositions of feces and cecal content show similarities within the groups, yet significant variations are observed in terms of richness and abundance between these two sample types, suggesting that fecal microbiota do not fully represent the abundance and composition of the gut microbiota. High-throughput sequencing validation supported the analysis of dominant taxonomic composition using DGGE, but it also revealed limitations in accurately assessing the alpha diversity of bacterial species.

## Data Availability

The paper and the supplemental materials present all the data needed to evaluate the conclusions. The raw data from the 16S metagenomics sequencing have been deposited in the NCBI Sequence Read Archive database under accession No. PRJNA315187. Additional data related to this paper may be requested from the corresponding author upon reasonable request.

## SUPPLEMENTAL MATERIAL

Supplemental material spectrum.04089-23-s0001.docx Tables S1 to S4.

ASM does not own the copyrights to Supplemental Material that may be linked to, or accessed through, an article. The authors have granted ASM a non-exclusive, world-wide license to publish the Supplemental Material files. Please contact the corresponding author directly for reuse.
