# Supplementary material for: Impact of whole grain highland hull-less barley on the denaturing gradient gel electrophoresis profiles of gut microbial communities in rats fed high-fat diets
Source: Microbiol Spectr. 2024 May 15;12(6):e04089-23. doi: 10.1128/spectrum.04089-23 (PMC11237377; doi:10.1128/spectrum.04089-23)
Supplement: Supplemental material — Tables S1 to S4. [file spectrum.04089-23-s0001.docx]

**Supplementary Tables**

**Table S1. Composition of experimental diets**

| **Ingredient (g/kg)** | **NC** | **BC** | **LD** | **HD** |
| --- | --- | --- | --- | --- |
| Corn starch | 529.50 | 489.50 | 389.50 | - |
| WHLB | - | - | 100.00 | 489.50 |
| Soybean oil | 70.00 | - | - | - |
| Lard | - | 100.00 | 100.00 | 100.00 |
| Cholesterol | - | 10.00 | 10.00 | 10.00 |
| Casein (99% protein) | 200.00 | 200.00 | 200.00 | 200.00 |
| Sucrose | 100.00 | 100.00 | 100.00 | 100.00 |
| Cellulose | 50.00 | 50.00 | 50.00 | 50.00 |
| L-cystine | 3.00 | 3.00 | 3.00 | 3.00 |
| Choline chloride | 2.50 | 2.50 | 2.50 | 2.50 |
| AIN-93G mineral mixture | 35.00 | 35.00 | 35.00 | 35.00 |
| AIN-93G vitamin mixture | 10.00 | 10.00 | 10.00 | 10.00 |
| Total | 1000.00 | 1000.00 | 1000.00 | 1000.00 |
| **Content (g/100g)** |  |  |  |  |
| Fat | 7.00 | 11.00 | 11.09 | 11.46 |
| Protein | 20.10 | 20.10 | 21.65 | 27.70 |
| β-glucan | 0.00 | 0.00 | 0.53 | 2.58 |
| Dietary fiber | 5.00 | 5.00 | 6.74 | 13.49 |

WHLB was dried at 55 °C for 24 h and then ground and passed through an 80-mesh sieve (0.5 mm). The diets were prepared weekly and stored at 4 °C. “-”: not added. Abbreviations: NC, normal control group; BC, blank control group; LD, low-dose group; HD, high-dose group; WHLB, whole-grain highland hull-less barley.

**Table S2. Formulation of 8% (w/v) polyacrylamide gel with different denaturant gradients**

| Reagent | 0% | 100% | 30% | 50% |
| --- | --- | --- | --- | --- |
| 40% Acrylamide/bis-acrylamide | 20.00 mL | 20.00 mL | 20.00 mL | 20.00 mL |
| 50 × TAE buffer | 2.00 mL | 2.00 mL | 2.00 mL | 2.00 mL |
| Deionized formamide | - | 40.00 mL | 12.00 mL | 20.00 mL |
| Urea | - | 42.00 g | 12.60 g | 21.00 g |
| Add dH_2_O to | 100.00 mL | 100.00 mL | 100.00 mL | 100.00 mL |

“-”: not added. After preparation, sonicated the gel for 15 minutes to de-gas it, filter it through a 0.45 μm aqueous phase filter membrane, and store it at 4 ℃ in the dark for no longer than one month. Add a final concentration of 0.09% (v/v) each of ammonium persulphate and N,N,N',N'-tetramethylenediamine solutions before casting the gel. 50 × TAE buffer: containing 2 M Tris base, 1 M sodium acetate, and 50 mM EDTA, pH 8.0.

**Table S3.** **Intensity of bands exhibited by adjusted volume**

| Band | No. | Group | | | | | | | | | | | | | | | |
| --- | --- | --- | --- | --- | --- | --- | --- | --- | --- | --- | --- | --- | --- | --- | --- | --- | --- |
|  |  | F-NC-W4 | F-NC-W8 | F-BC-W4 | F-BC-W8 | F-LD-W4 | F-LD-W8 | F-HD-W4 | F-HD-W8 | C-NC-W4 | C-NC-W8 | C-BC-W4 | C-BC-W8 | C-LD-W4 | C-LD-W8 | C-HD-W4 | C-HD-W8 |
| F-1 | 1 | **-** | **-** | **-** | **-** | **-** | **-** | **-** | **-** | 6594 | 17262 | 35070 | 13902 | 18144 | 14322 | 39144 | 49770 |
| FC-1 | 2 | 1640 | 31160 | 14440 | 37560 | 33040 | 8840 | 71720 | 70240 | 49476 | 105084 | 78540 | 6342 | 48342 | 33348 | 53760 | 21000 |
| C-1 | 3 | 15560 | 50400 | 56200 | 32360 | 8520 | 11160 | 4400 | 9360 | **-** | **-** | **-** | **-** | **-** | **-** | **-** | **-** |
| FC-2 | 4 | 25480 | 62080 | 35080 | 56360 | 13800 | 44960 | 20840 | 30080 | 30576 | 67032 | 30870 | 15792 | 24276 | 8022 | 21210 | 16338 |
| FC-3 | 5 | 4600 | 20880 | 7000 | 5080 | 8960 | 15920 | 27960 | 11240 | 9198 | 8862 | 13524 | 19824 | 20790 | 23814 | 54348 | 5796 |
| FC-4 | 6 | 7800 | 17720 | 13880 | 10240 | 23080 | 12760 | 20640 | 14920 | 8526 | 43596 | 12096 | 3108 | 27006 | 2856 | 8778 | 1428 |
| FC-5 | 7 | 45760 | 18720 | 47680 | 6760 | 33920 | 22440 | 10600 | 23240 | 38220 | 3990 | 12180 | 49140 | 9576 | 16170 | 11046 | 17724 |
| C-2 | 8 | 1600 | 17440 | 4480 | 12920 | 6000 | 15040 | 15160 | 600 | **-** | **-** | **-** | **-** | **-** | **-** | **-** | **-** |
| FC-6 | 9 | 7320 | 22400 | 20360 | 33400 | 25760 | 24960 | 4160 | 18840 | 18270 | 6216 | 10332 | 13902 | 37506 | 23100 | 252 | 8904 |
| FC-7 | 10 | 3200 | 20880 | 1800 | 4560 | 18120 | 10160 | 14960 | 20840 | 14196 | 7980 | 10542 | 9072 | 43260 | 20748 | 5544 | 1806 |
| FC-8 | 11 | 28760 | 11840 | 15320 | 11000 | 26520 | 20560 | 24840 | 48800 | 33894 | 34818 | 12852 | 29484 | 41202 | 24066 | 26166 | 9870 |
| FC-9 | 12 | 29160 | 10440 | 8800 | 7640 | 32040 | 19000 | 12160 | 13160 | 18858 | 6846 | 9828 | 31542 | 10584 | 16128 | 14490 | 7056 |
| C-3 | 13 | 11920 | 11600 | 4640 | 18840 | 7200 | 33160 | 6520 | 9280 | **-** | **-** | **-** | **-** | **-** | **-** | **-** | **-** |
| FC-10 | 14 | 7440 | 38520 | 9040 | 15320 | 10600 | 9200 | 17160 | 28640 | 37086 | 75768 | 13230 | 17178 | 39480 | 41874 | 75138 | 36876 |
| C-4 | 15 | 13000 | 11360 | 2720 | 23760 | 13280 | 9320 | 13800 | 48520 | **-** | **-** | **-** | **-** | **-** | **-** | **-** | **-** |
| FC-11 | 16 | 27760 | 14040 | 8920 | 16160 | 49400 | 21600 | 40840 | 50760 | 20538 | 18564 | 13188 | 26628 | 40572 | 40488 | 33642 | 20412 |
| FC-12 | 17 | 20880 | 3400 | 15840 | 5520 | 69640 | 20320 | 6840 | 23400 | 39270 | 30912 | 17094 | 31542 | 53970 | 22176 | 12516 | 2478 |
| C-5 | 18 | 17720 | 20640 | 44440 | 35120 | 15920 | 36720 | 21360 | 14120 | **-** | **-** | **-** | **-** | **-** | **-** | **-** | **-** |
| FC-13 | 19 | 18400 | 47160 | 21440 | 24800 | 12520 | 32280 | 54720 | 33840 | 33768 | 38598 | 13482 | 23310 | 33138 | 22134 | 49686 | 12054 |
| FC-14 | 20 | 28400 | 5480 | 3160 | 2400 | 7920 | 7040 | 6640 | 12880 | 33432 | 1512 | 11550 | 6720 | 12936 | 4536 | 1260 | 4158 |
| FC-15 | 21 | 24680 | 16160 | 21800 | 35960 | 23520 | 18280 | 11000 | 24640 | 12936 | 9786 | 22512 | 54600 | 6762 | 4158 | 10542 | 2478 |

“-”: not detected.

**Table S4. Sequence identities to the closest relatives of sequences obtained from DGGE profiles**

| Band No. | BLAST result | Sequence size  （bp） | Identity | GenBank accession No. |
| --- | --- | --- | --- | --- |
| F-1 | *Lactobacillus gasseri* | 165 | 97% | GU417972.1 |
| FC-1 | Uncultured *Prevotella* sp. | 162 | 98% | KP106487.1 |
| FC-2 | Uncultured *Phascolarctobacterium* sp. | 169 | 97% | KP108154.1 |
| FC-3 | Uncultured *Clostridium* sp. | 152 | 99% | KP106011.1 |
| FC-4 | Uncultured *Acetivibrio* sp. | 148 | 99% | KP109424.1 |
| FC-5 | *Bacteroides* sp. | 163 | 97% | JX519759.1 |
| FC-6 | *Ruminococcus* sp. | 144 | 99% | KP114244.1 |
| FC-7 | *Clostridium* sp. | 143 | 98% | LN868251.1 |
| FC-8 | Uncultured Bacteroidetes bacterium | 164 | 96% | GU959060.1 |
| FC-9 | Uncultured *Prevotella* sp. | 163 | 95% | KP104139.1 |
| FC-10 | Uncultured bacterium | 144 | 98% | KC310428.1 |
| FC-11 | *Clostridium* sp. | 151 | 98% | AB622849.1 |
| FC-12 | Uncultured *Anaerovibrio* sp. | 169 | 96% | KP104404.1 |
| FC-13 | Uncultured Ruminococcaceae bacterium | 156 | 85% | KP107424.1 |
| FC-14 | Uncultured Bacteroidetes bacterium | 160 | 98% | GU958752.1 |
| FC-15 | Uncultured Firmicutes bacterium | 145 | 99% | GU959535.1 |
| C-1 | Uncultured *Barnesiella* sp. | 150 | 97% | KP101710.1 |
| C-2 | Uncultured Bacteroidetes bacterium | 162 | 97% | GU958246.1 |
| C-3 | Uncultured bacterium | 148 | 99% | GQ225198.1 |
| C-4 | Uncultured Lachnospiraceae bacterium | 146 | 100% | KP102868.1 |
| C-5 | *Ruminococcus* sp. | 153 | 99% | GU324399.1 |
